# Prevention of Abdominal Bulging Using Onlay Dermal Autografts from Discarded Zone IV TRAM Flap Tissue

**DOI:** 10.3390/jcm11071929

**Published:** 2022-03-30

**Authors:** Won Seob Lee, Seong Oh Park, Il-Kug Kim

**Affiliations:** 1 Department of Plastic and Reconstructive Surgery, Yeungnam University College of Medicine, Daegu 42415, Korea; twinreo6542@ynu.ac.kr; 2 Department of Plastic and Reconstructive Surgery, Hanyang University College of Medicine, Seoul 04763, Korea

**Keywords:** breast reconstruction, tissue donors, dermis graft, abdominal hernia, complications

## Abstract

While the transverse rectus abdominis myocutaneous (TRAM) flap is a popular option for abdominal-based breast reconstruction, abdominal wall morbidities such as bulging or hernia remain a concern. Here, we introduced a surgical technique for reinforcing the abdominal wall using an onlay autograft obtained from discarded zone IV tissue following a primary closure. We compared abdominal wall morbidities between patients receiving an onlay graft and those receiving primary closure only. We retrospectively reviewed the medical charts of patients who underwent breast reconstruction using a TRAM flap between December 2018 and May 2021. Additionally, we assessed donor-site morbidities based on physical examination. Of the 79 patients included, 38 had received a dermal graft and 41 had not. Donor-site morbidities occurred in 10 (24.5%) and 1 (2.6%) patients, and bulging occurred in 8 (19.5%) and 1 (2.6%) patients in the primary closure and dermal autograft groups, respectively. A statistically significant difference in the incidence of bulging was observed between the groups (*p* = 0.030). In conclusion, the introduction of a dermal autograft after primary closure can successfully ameliorate morbidities at the TRAM flap site.

## 1. Introduction

Abdominal-based breast reconstruction is widely applied to achieve a natural breast shape, volume, and contour using autologous tissues after a mastectomy [1]. Contrary to smaller tissue sources such as the latissimus dorsi myocutaneous flap, the abdomen offers enough tissue and thus no synthetic materials are needed. Since 1979, when Holmström first introduced autologous breast reconstruction using lower abdominal tissues, several surgical techniques have been developed [2,3,4]. These involve free transverse rectus abdominis myocutaneous (TRAM) flaps, muscle-sparing TRAM flaps, deep inferior epigastric perforator (DIEP) flaps, or superficial inferior epigastric artery (SIEA) flaps. Nowadays, many surgeons opt for these techniques to achieve satisfactory and safe outcomes [5,6,7].

When using a TRAM flap, muscle dissection during flap elevation is unavoidable; however, improperly managed abdominal wall defects may result in a hernia or bulging. These abnormalities in the lower abdomen are likely owed to the thin and fragile architecture of the posterior rectus which bears a single layer of fascia below the arcuate line. Postoperative complications such as abdominal bulge, epigastric fullness, and hernia have been reported in 0–35% of cases [8,9,10,11].

A consensus on how to properly manage abdominal wall defects has yet to be reached. Abdominal wall closure techniques such as direct closure [12], the use of synthetic meshes, or acellular dermal matrixes (ADM) [13,14] all bear substantial limitations. Direct closure can often lead to the recurrence of seroma, whereas synthetic meshes often cause infections or foreign body reactions [15,16]. In addition to being costly, ADM may exhibit limited tissue integration, local complications, or hernia due to chronic infection or a foreign body reaction [17]. With ADM prices of USD 40/cm^2^, an 8 × 16 cm^2^ ADM raises the cost to USD 5000. Meanwhile, dermal autografts may excellently bypass these limitations since autologous tissues are easier to harvest, cheaper, and less likely to cause such complications. In fact, several studies have endorsed the usage of dermal grafts for avoiding donor-site morbidities [18,19].

Here, we use a surgical technique to reinforce the abdominal wall by means of a dermal onlay autograft from zone IV tissue discarded after a primary closure. Additionally, we study the incidence of donor-site morbidities such as abdominal budging between patients receiving an onlay graft and those being subjected only to primary closure.

## 2. Materials and Methods

### 2.1. Patients and Clinical Data Collection

The study population included 79 females who underwent breast reconstruction with a TRAM flap between December 2018 and May 2021. We performed free TRAM flap instead of DIEP flap when the perforator with more than 2 mm in diameter was not observed in the preoperative CT angiography. If the patient had a fear of microvascular anastomosis failure, a pedicled TRAM flap was performed instead of free flap. We specifically excluded patients who had undergone bilateral breast reconstruction were excluded because primary closure of the abdominal fascia is not possible. The patients were divided into two groups based on the technique applied: a primary closure (PC) only group and a dermal autograft following primary closure (PC + DA) group. Data including age, body mass index (BMI), follow-up period, smoking status, anticoagulant use, chemotherapy (CTx) use, radiotherapy (RTx) use, history of abdominal surgery, comorbidities (diabetes mellitus [DM], hypertension [HTN], dyslipidemia), timing of reconstruction, type of reconstruction, and donor morbidity were collected from the patients’ medical records. Patients were followed up to one, three, and six months after surgery. After six months, patients were monitored at six-month intervals. Abdominal bulging was assessed by physical examination and according to the authors’ experience. Cases with abdominal bulging were all classified as bulging based on the presence of fascia defects upon radiological examination [20]. No indications of hernia were found for any of the 79 patients.

### 2.2. Surgical Technique

A TRAM flap with a standard elliptical design was used to perform flap elevation [21]. It bore minimal amounts of rectus abdominis fascia to ensure that the fascia remained almost intact. To inset the elevated abdominal flap into the breast pocket, the flap was trimmed to an appropriate volume taking the preoperative assessment and the weight and volume of the mastectomized breast tissue into account. Then, only the dermis was carefully harvested from the discarded flap (parts of zone IV and II) and prepared for downstream use.

**Figure 1:**
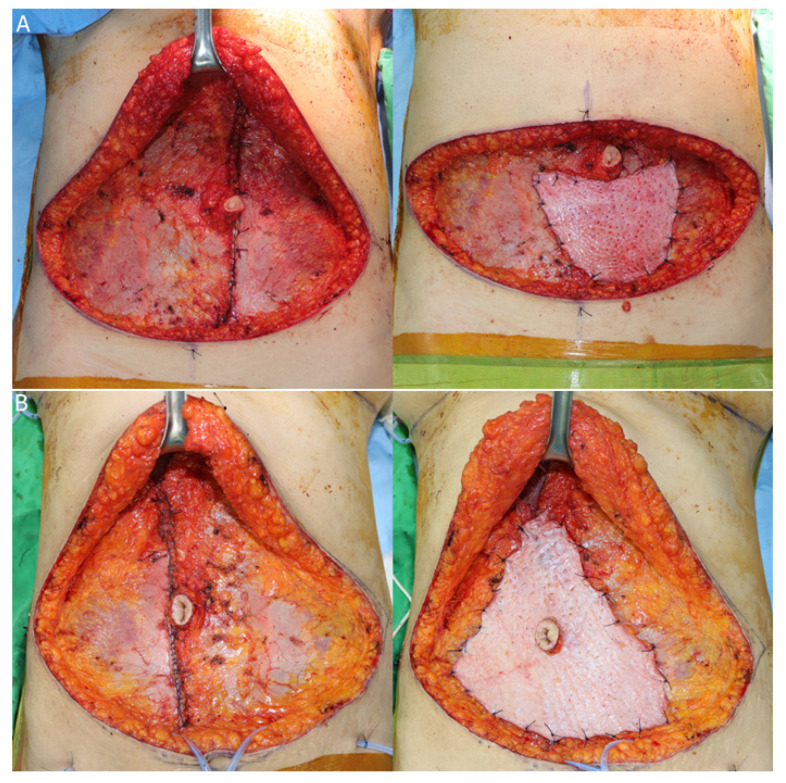
Onlay dermal autograft from discarded zone IV tissue in the TRAM flap: (**A**) Direct closure was performed on both sides of the fascia in a horizontal-mattress fashion following flap elevation. The dermal onlay graft harvested from the discarded flap was stretched over the fascia on which the direct closure was performed. (**B**) An incision was made on the dermis at the site of the umbilicus through which the umbilical stalk was passed.

Τhe abdominal wall defect from which the flap had been harvested was sewn on both sides of the fascia in a horizontal-mattress fashion. After complete closure, the prepared dermal onlay autograft was placed over the fascia. The basement membrane of the dermis came in contact with the fascia after closure, and grafting was implemented by stretching the autograft as much as possible over the area below the arcuate line. An incision was made on the dermis at the site of overlap with the umbilicus through which the umbilical stalk was passed (Figure 1).

### 2.3. Statistical Analysis

Statistical analyses were performed using the SPSS statistical software version 22 (IBM Corp., Armonk, NY, USA). The differences between groups were assessed using an independent two-sample *t*-test for age, BMI, and follow-up period. A one-tailed Fisher’s exact test was performed for smoking status, anticoagulant use, CTx, RTx, history of abdominal surgery, comorbidities (DM, HTN, dyslipidemia), time of surgery, method of surgery, and donor morbidity. Statistical significance was set to *p* < 0.05.

## 3. Results

The study population consisted of a total of 79 patients (38 in the PC+DA group and 41 in the PC-only group), and a summary of patients’ demographics is provided in Table 1. The mean age and follow-up period were 48.5 ± 7.4 and 1.24 ± 0.62 years in the PC group and 47.9 ± 7.5 and 1.04 ± 0.37 years in the PC + DA group, respectively. Age, follow-up period, smoking status, anticoagulant use, CTx, RTx, history of abdominal surgery, and comorbidities (DM, HTN, dyslipidemia) did not differ significantly between the two groups. In contrast, the mean BMI was significantly different in the PC group (23.2 ± 3.0 kg/m^2^) compared with the PC+DA group (25.0 ± 3.3 kg/m^2^; *p* = 0.011).

With respect to operative characteristics, there was no significant difference in the time of surgery reconstruction between the two groups. In the PC group, free TRAM had been used in the majority (63.4%) of the cases, whereas in the PC + DA group, pedicled TRAM flaps had been used in almost all cases (92.1%). There was a statistically significant difference in the method of surgery between the groups (*p* < 0.01) (Table 2).

Donor-site complications were found in 10 patients in the PC group, whereas only one case bore a donor-site complication in the PC + DA group. Of those, eight could be classified as bulging (19.5%) in the PC group and the one positive case in the PC + DA group accounted for 2.6%. There was a statistically significant difference in the incidence of bulging between the two groups (*p* = 0.030; Table 3).

**Figure 2:**
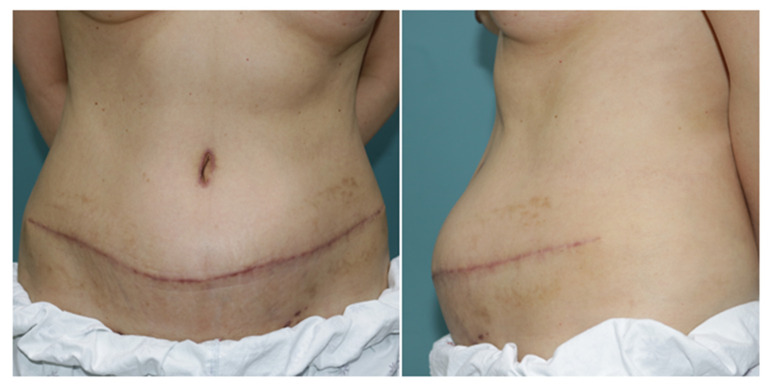
Clinical image of a 50-year-old female patient taken 6 months after delayed breast reconstruction with a pedicled TRAM flap. Direct closure was performed on the donor's fascial defect. A distinct bulging at the site of the fascial defect was found during a physical examination.

**Figure 3:**
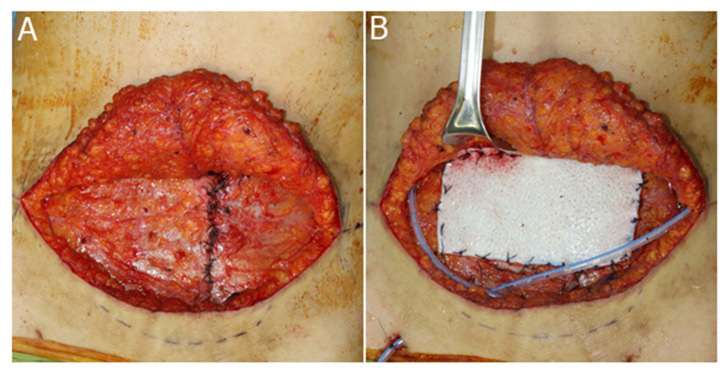
Surgical techniques used for the correction of abdominal bulging. (**A**) Plication of the abdominal fascia. (**B**) Onlay graft of acellular dermal matrix.

Among the eight patients who exhibited bulging in the PC group, two patients had moderate bulging which required revision surgery. These two patients visited our hospital due to lower abdominal bulging that occurred with a delay of six months after breast reconstruction with free TRAM flaps (Figure 2 and Figure 3). A hernia was not observed on computed tomography (CT), but moderate bulging with contour irregularities was observed. During the revision operation, an overall thinning devoid of fascial defects were observed in the abdominal wall. We reinforced the latter with an acellular dermal matrix following fascia plication (Figure 3). We conclude that an overall fascial reinforcement may be as important as a defect reinforcement.

## 4. Discussion

We investigated the effect of dermal onlay autografts in reducing donor-site morbidity. We evaluated the incidence of complications by comparing a PC-only group and a PC + DA group. As a result, the incidence of complications in the PC + DA group was lower than in the PC group (24.4% vs. 2.6%). For bulging, the incidence was less in the PC + DA group than in the PC group (19.5% vs. 2.6%) and was statistically significant (*p* = 0.030). This suggests that, upon DA application, complications are less likely to appear.

TRAM flaps have been used extensively for breast reconstruction, but donor-related morbidities have persistently concerned surgeons. These can be ameliorated by minimizing the extent of each defect during flap elevation or by reconstructing the abdominal wall. In the first case, advanced flap elevation methods against muscle or fascial defects such as the DIEP flap have been deployed [9,12]. For example, to maximize pedicle length with negligible abdominal wall damage, robot-assisted harvesting of DIEP flaps was introduced recently [22]. However, despite extensive research, no consensus has been reached on the second method. In 1987, Hartrampf and Bennett performed muscle-sparing TRAM flap surgery and emphasized the importance of two-layered closure for both internal and external oblique muscle fascia [2]. In cases with inadequate tension or difficulty with primary closure, they used a mesh with appropriate tension to achieve coverage. Meanwhile, Park et al. introduced a method of primary closure with W-plasty for evenly distributing tension on the fascia [23]. Ascherman et al. reinforced the abdominal wall using a prolene mesh for onlay grafts routinely after a primary closure [7]. Bharti et al. introduced grafts with folded meshes to cover fascia defects following bi-pedicled TRAM flap procedures [24]. While these techniques have contributed to reducing donor-site morbidities, the prevalence of the latter still remains significantly high [25]. A synthetic mesh may cause additional complications, such as infection, foreign body reaction, and extrusion [15,16]. ADM can also provide structural strength and is often applied for breast reconstruction using an implant [26,27]. Although ADM has been a popular choice in recent years for reducing donor-site morbidities associated with the TRAM flap, not only is it not superior to the aforementioned methods but also a costly option [17].

In that respect, dermal autografts cause fewer complications than synthetic materials. The autologous dermis is elastic and biologically active [28]. Moreover, the connective tissue fibers within the dermis cause inflammation which results in revascularization, fibroblast proliferation, and collagen increase [19]. Such processes render complications less likely to occur and explain why the dermis is abundantly used in many fields.

Reinforcement of abdominal wall defects using a flap discarded from previous surgical procedures was first introduced by Loewe in 1913 [29]. Hein et al. performed abdominal fascial repair in 24 patients between 1995 and 1997 using a dermal graft [15]. Kheradmand et al. reported similar complication rates resulting from dermal grafts from discarded flaps and from mesh grafts for fascia defects [19]. We used a dermal onlay autograft following a direct closure of the fascia. With minimal inclusion of the rectus abdominis fascia during flap elevation, we achieved sufficient direct closure with little tension in all patients treated. Inappropriate inlay grafts may result in bulging due to the loosening of the reconstructed abdominal wall and the insufficient tension applied. To avoid this, we chose a method that can effectively maintain appropriate tension through direct closure, while sufficiently reinforcing the abdominal wall with the dermal graft. Compared to the incidence of bulging in 19.5% of cases with direct closure alone, our technique showed a pronounced reduction with only 2.6% of cases bearing complications. Such findings are comparable to those of other studies. For example, Kheradmand et al. found no significant differences in the incidence of hernia and bulging between a dermal graft and a mesh group (2.9% vs. 2.3%, respectively) [19]. Similarly, Park et al. reported 2.1% of hernia cases after fascia-sparing and reinforcement below the arcuate line using remnants of the caudal rectus muscle [23].

We combined primary closure and onlay grafting to benefit from the advantages of both techniques [30]. We chose the onlay graft method as it allows for more precise and delicate graft integration and attachment [7]. An onlay graft diminishes the defects, even in the relatively vulnerable areas below the arcuate line or on the suture line. In addition, the graft may be used for the overall reinforcement of the fascia. Mizgala et al. and Lejour and Dome reported that abdominal muscle strength decreases by approximately 45% over time after the application of TRAM flaps [31,32]. Such thinning of the fascia after direct closure of the donor site over time requires reinforcing not only the site of the defect but also the entire fascia.

In the present study, the free TRAM flap was most commonly used in the PC group (63.4%), whereas the pedicled one was used in 92.1% of the PC + DA cases. We argue that a pedicled TRAM flap may cause greater damage to the abdominal wall as, during flap elevation, pedicled TRAM flaps have been shown to damage the rectus abdominis muscle more than free TRAM flaps alongside triggering complications and challenging the repair of a donor-site hernia [33,34]. Even if the abdominal wall is somewhat damaged during surgery, the dermal graft is expected to prevent bulging, in line with our findings that dermal grafts prevent bulging as efficiently as pedicled TRAM flaps.

In the present study, revision surgery was required in two cases with complications in the PC group. The patients were admitted to our hospital with a major complaint of lower abdominal bulging that occurred approximately six months after surgery (Figure 2). Although CT findings showed no signs of hernia, they presented moderate bulging with contour irregularity. During the revision operation, overall thinning of the abdominal wall was observed, but there were no fascial defects. We reinforced the abdominal wall with an acellular dermal matrix following fascial plication. These findings underscore why a reinforcement of the entire fascia, and not just the defect, is important.

The present study bears certain limitations. First, a selection bias may have been introduced by the retrospective nature of the study. Second, bulging was subjectively assessed by physicians as mild, moderate, or severe whereas other complications were assessed merely from clinical data. Third, our sample size was relatively small. Aside from bulging, no other complications occurred to allow a comparison between the two groups. To verify these results and conclude about the missing parameters, a larger-scale prospective study will be needed.

## 5. Conclusions

Dermal onlay autografts bear certain advantages over conventional flaps for managing abdominal wall defects including the limited recurrence of donor-site complications and the lower risk of infections or foreign body reactions. Moreover, because a flap from discarded tissue is used, it is easier and cheaper to obtain it while allowing for a delicate and stronger reinforcement of the desired site. In conclusion, the surgical technique introduced in this study could be considered effective for reducing donor-site morbidities following TRAM flap.

## Figures and Tables

**Table 1:** Patients’ demographics.

	Primary Closure (%)	Dermal Autograft (%)	*p*
No.	41	38	
Mean age ± SD	48.5 ± 7.4	47.9 ± 7.5	0.759
BMI ± SD	23.2 ± 3.0	25 ± 3.3	0.011 †
Follow-up period ± SD	1.24 ± 0.62	1.04 ± 0.37	0.084
Smokers	3 (7.3)	2 (5.3)	1.000
Anticoagulant use	1 (2.4)	1 (2.6)	1.000
History of abdominal surgery	4 (9.8)	3 (7.9)	1.000
Chemotherapy	10 (24.4)	12 (31.6)	0.616
Radiotherapy	5 (12.2)	4 (10.5)	1.000
Comorbidity			
Diabetes mellitus	3 (7.3)	5 (13.2)	0.471
Hypertension	3 (7.3)	7 (18.4)	0.183
Dyslipidemia	2 (4.9)	5 (13.2)	0.252

BMI, body mass index; SD, standard deviation; N/A, not applicable. † *p* < 0.05.

**Table 2:** Operative characteristics.

	Primary Closure (%)	Dermal Autograft (%)	*p*
Time of reconstruction			0.133
Immediate	27 (65.9)	31 (81.6)	
Delayed	14 (34.1)	7 (18.4)	
Type of reconstruction			<0.05 †
Free TRAM	26 (63.4)	3 (7.9)	
Pedicled TRAM	15 (36.6)	35 (92.1)	

TRAM, transverse rectus abdominus myocutaneous. † *p* < 0.05.

**Table 3:** Donor-site morbidities.

	Primary Closure (%)	Dermal Autograft (%)	*p*
Donor Morbidity			
Bulging	8 (19.5)	1 (2.6)	0.030 †
Hernia	0 (0.0)	0 (0.0)	N/A
Fat necrosis	1 (2.4)	0 (0.0)	1.000
Seroma	0 (0.0)	0 (0.0)	N/A
Umbilicus necrosis	1 (2.4)	0 (0.0)	1.000
Wound dehiscence	0 (0.0)	0 (0.0)	N/A

N/A, not applicable. † *p* < 0.05.

## Data Availability

The data presented in this study are available on request from the corresponding author. The data are not publicly available due to consideration for the patients’ privacy.
